# Imaging of the Lamina Cribrosa using Swept-Source Optical Coherence Tomography

**DOI:** 10.5005/jp-journals-10008-1117

**Published:** 2012-10-16

**Authors:** Brenda Nuyen, Kaweh Mansouri, Robert N Weinreb

**Affiliations:** Department of Ophthalmology, Hamilton Glaucoma Center and Shiley Eye Center, University of California, San Diego, La Jolla, California, USA; Research Fellow, Department of Ophthalmology, Hamilton Glaucoma Center and Shiley Eye Center, University of California, San Diego, La Jolla, California, USA; Chairman, Department of Ophthalmology, Director, Hamilton Glaucoma Center and Shiley Eye Center, University of California, San Diego, La Jolla, California, USA

**Keywords:** Glaucoma, Intraocular pressure, Swept-source optical coherence tomography.

## Abstract

The lamina cribrosa (LC) is the presumed site of axonal injury in glaucoma. Its deformation has been suggested to contribute to optic neuropathy by impeding axoplasmic flow within the optic nerve fibers, leading to apoptosis of retinal ganglion cells. To visualize the LC *in vivo*, optical coherence tomography (OCT) has been applied. Spectral domain (SD)-OCT, used in conjunction with recently introduced enhanced depth imaging (EDI)-OCT, has improved visualization of deeper ocular layers, but in many individuals it is still limited by inadequate resolution, poor image contrast and insufficient depth penetrance. The posterior laminar surface especially is not viewed clearly using these methods. New generation high-penetration (HP)-OCTs, also known as swept-source (SS)-OCT, are capable to evaluate the choroid *in vivo* to a remarkable level of detail. SS-OCTs use a longer wavelength (1,050 nm instead of 840 nm) compared to the conventional techniques. We review current knowledge of the LC, findings from trials that use SD-OCT and EDI-OCT, and our experience with a prototype SS-OCT to visualize the LC in its entirety.

Key Points

*What is known*?

•     The LC is the presumed site of axonal injury in glaucoma

•     Compared to spectral domain-OCT, enhanced depth imaging-OCT improves imaging of the LC

•     Even so, currently used SD-OCT techniques are restricted by poor wavelength penetrance of the deeper ocular layers

*What our findings add*?

•    SS-OCT may be a superior imaging modality for deep ocular structures

•    Prior studies used SS-OCT to evaluate choroidal thickness in both healthy and ‘normal tension glaucoma’ eyes

•    SS-OCT enables good evaluation of three-dimension (3D) lamina cribrosa morphology.

**How to cite this article:** Nuyen B, Mansouri K, Weinreb RN. Imaging of the Lamina Cribrosa using Swept-Source Optical Coherence Tomography. J Current Glau Prac 2012;6(3): 113-119.

## INTRODUCTION

A leading cause of blindness worldwide,^1^ glaucoma comprises a group of progressive optic neuropathies with characteristic optic disk damage and a concomitant pattern of visual field loss.^2^ This disease affects more than 66 million people worldwide, and in the United States, more than 7 million office visits occur every year to monitor patients who have or are at risk of developing glaucoma.^3,4^ Furthermore, a substantial number of individuals remain undiagnosed or inadequately treated, and the severity of this problem in terms of morbidity and health care resources will only escalate as the population becomes older.^5^ Although much has been done to study risk factors and mechanisms, the basic pathophysiology of the disease remains poorly understood.

The axons of the retinal ganglion cells constitute the retinal nerve fiber layer (RNFL), the innermost layer of the retina. The convergence of the axons forms the neuroretinal rim of the nerve before exiting the eye through the lamina cribrosa (LC), a scleral structure that is characterized by sheets of porous connective tissue. It is believed that the LC provides mechanical support to optic nerve fibers within the deep optic disk region.^6^ Trophic factors, such as brain-derived neurotrophic factor, are transported in both an anterograde and retrograde fashion via the axons of the retinal ganglion cells through the LC, to their cell bodies and to the lateral geniculate nucleus.^2^ These substances are essential to the survival of these neurons.

Glaucoma is characterized by the progressive degeneration of the retinal ganglion cells, axons, soma and dendrites.^7,8^ On the basis of histopathologic studies, the LC has been implicated as the site of original optic nerve damage in glaucoma.^9^ Changes in the LC, especially deformation and compression, have been related to a decrease in connective tissue, resulting in structural thinning that is linked to glaucomatous axonal damage.^10,11^ Morphological changes in the LC pore shape and size also have been correlated with the severity and progression of glaucoma.^12-14^ In monkey eyes, chronic ocular hypertension has been shown to result in particular changes in the LC, including outward migration of the posterior lamina insertion point.^6^ Overall, the structural thinning, pore deformities and posterior displacement of the LC cause the pores to deform.^15^ This deformation likely impedes axoplasmic flow and disrupts transport of trophic factors important to the survival of retinal ganglion cells.^16,17^ Thus, structural changes in the LC may play a role in neuronal death characteristic of glaucoma. Interestingly, from a biomechanical standpoint, the LC also represents a discontinuity in the spherical casing of the eye, which makes it more vulnerable to the mechanical stress loading that may play a role in glaucoma.^18^ Understanding the forces that regulate parameters of the LC will further elucidate the pathophysiology of glaucoma. Some investigators have suggested that characterization of focal defects, in addition to general morphologic changes, may provide valuable insight into specific glaucomatous mechanisms at this site of presumed axonal injury.^19^ A detailed study of the LC, however, requires accurate visualization of this anatomical structure.

In the past, limited visualization of the LC on clinical examination has made its evaluation challenging to the extent that most available information about this structure has been gathered from postmortem analysis of human eyes with glaucoma^20,21^ or animal eyes with experimental glaucoma.^22,23^ Such studies are prone to the distorting effects of fixation, and it is largely unknown how changes in IOP affect enucleated eyes.^20,24,25^ Thus, *in vivo* characterization of the LC, including features such as its thickness and average pore shape and size, is an essential part of studying glaucomatous disease.

## OPTICAL COHERENCE TOMOGRAPHY FOR VISUALIZATION OF THE LC

### Spectral Domain-OCT

Time-domain optical coherence tomography (OCT) reveals the LC as a region of high back reflection underneath the optic disk cup, which does not allow for precise characterization of the structure.^26^ The development of spectral domain (SD)-OCT has heralded improved assessment of the LC.^27-29^ A main limitation is the fact that the anterior and especially posterior borders of the LC are not sharply delineated when viewed using this OCT method, which impedes the accuracy of characterization.

### Enhanced Depth Imaging-OCT

Enhanced depth imaging (EDI)-OCT was developed by Spaide et al in 2008 as a superior imaging method of deeper ocular structures.^30-36^ In EDI-OCT, the instrument is placed close enough to the eye to create an inverted representation of the fundus, which increases the sensitivity of imaging of the deeper layers. Frequently the Heidelberg Spectralis (Heidelberg Engineering, Heidelberg, Germany) is used, although other SD-OCT instruments have also introduced EDI ability.^37^ The deeper portions of the choroid and inner sclera are placed closer to the zero delay, so that these structures have a smaller frequency.^37^ With this method, *in vivo* imaging sensitivity of the deep layers is enhanced.^37^ Yet EDI-OCT usually involves multiple averaging to achieve high contrast and low noise, which still produces difficulties with image detail and high scattering, particularly with the posterior choroid and sclera.^38-40^ With EDI-OCT, the deeper portion and posterior border of the LC lack the clarity required for precise characterization of the structure.^41^

EDI-OCT was used initially in retina research to characterize features of the choroid in healthy and diseased eyes, investigating features, such as including thickness, volume, degree of calcification and presence of cavitation.^42-45^ Only recently has research turned toward the use of EDI-OCT to evaluate deep optic nerve structures. Park et al^41^ conducted a pilot study to assess the usefulness of EDI-OCT in evaluating the LC, accompanying vascular structures, and peripapillary choroid and sclera. In their EDI-OCT images, the LC was noticeably distinguished from surrounding tissue, especially in the central area. With previous OCT techniques, the anterior laminar surface could not be visualized beneath the neuroretinal rim, vascular structures or scleral rim.^37^ With EDI-OCT, the anterior surface could be at least partly traced even beneath those structures in 65% of the evaluated eyes. The size and direction of the pores in the LC were also determined in 76% of eyes. Furthermore, the EDI-OCT images demonstrated the trajectories of various vascular structures, such as the short posterior ciliary artery (in 86% of eyes), as well as the subarachnoid space (in 18% of eyes). They further demonstrated that EDI-OCT can provide satisfactory *in vivo* cross-sectional images of the LC and other deep optic nerve structures. However, a major limitation of EDI-OCT is that it is capable of visualizing the posterior laminar surface only in a minority of eyes.

EDI-OCT has also been used to compare differences in LC thickness between so-called ‘normal-tension glaucoma’ (NTG) and primary open-angle glaucoma (POAG). Park et al^26^ determined that laminar thickness in POAG patients was reduced relative to NTG patients with similar disease severity. Moreover, they reported that EDI-OCT imaging fared better than standard SD-OCT in terms of intraobserver, interobserver, intravisit and intervisit reproducibility (ICC = 0.966, ICC = 0.935, ICC = 0.956, ICC = 0.950 respectively). The neuroretinal and scleral rims obscured many sections of the LC and the thickness was thus measured only at the central laminar area.

EDI-OCT also has been used to construct and analyze *in situ* three-dimensional (3D) images of the LC in patients with POAG. Lee et al^46^ assessed the 3D configuration of the LC by two different techniques called ‘maximum intensity projection’ (MIP) and ‘texture-based volume rendering’ (VRT). MIP is a 3D image processing method that uses maximum or minimum intensity along one view. The VRT method uses a color map that provides visualization of highly reflective tissue, such as the LC, among a semitransparent view that represents low reflective tissue. Using the 3D rendering and the B-scans, the maximal and minimal LC depths were measured and compared. The depth calculated from the 3D images corresponded very well with that determined on the B-scan images (ICC = 1.0 between MIP and B-scan, 0.99 between VRT and B-scan). In addition, the 3D images were able to demonstrate variations in the LC shape that horizontal sections did not show. The authors concluded that evaluation of the 3D architecture of the laminar region may be a useful tool for early diagnosis of glaucoma and may be able to identify patients who are at greater risk of developing glaucoma. They reported limitations in visualization of deeper LC layers. Notably, the VRT image (more easily evaluated compared to that obtained by MIP) did not allow the visualization of the complete lamina, which was influenced by the suprajacent neuroretinal rim.

Work has also been done to evaluate the configuration and location of focal defects of the LC in glaucoma, and to correlate these parameters with accompanying visual field deficits.^19^ SD-OCT does describe general structural changes, such as thinning and outward migration, of the LC in glaucoma, but visualization of localized irregularities in the laminar surface would illustrate more specific mechanisms of glaucomatous injury. Kiumehr et al^19^ defined a focal LC defect as an anterior laminar surface irregularity of specific size and dimension that breaches the smooth curvilinear U- or W-shaped contour that is observed in nonglauco-matous eyes. They did not observe any feature fitting this definition in healthy eyes. The study found that the observed focal defects seen in EDI-OCT images correlated well with visual field mean deviation (ρ = -0.498, p = 0.003). As with the previous work described here,^41,46^ limitations of this study also revolved around the difficulty of full visualization of the LC. It should be noted that healthy eyes have a thicker neuroretinal rim, which may influence image scattering, and reduce the ability to visualize LC aspects in detail.

### Swept-Source-OCT

Most recently, high-penetration (HP)-OCT, also known as swept-source (SS)-OCT, has been developed and promises to enhance visualization of deeper ocular layers, including the choroid and LC.^38^ Higher penetration of the OCT probe light, which uses a center wavelength of approximately 1,050 nm instead of 840 nm (the wavelength used by current SD-OCT instruments), allows for deeper choroidal and even scleral imaging.^47-53^ With a longer wavelength, this method has less scattering at the photoreceptors and retinal pigment epithelium.^54^ Currently used for research purposes only, SS-OCT promises to provide a clearer image and more accurate characterization of the LC. We describe the SS-OCT method and report our findings using this technique to visualize and characterize the LC in its entirety.

Ikuno et al^40^ used a prototype SS-OCT to determine baseline choroidal thickness in healthy Japanese subjects. Mean choroidal thicknesses were approximately 354 μm at the fovea, 364 μm superiorly, 345 μm inferiorly, 227 μm nasally and 337 μm temporally; similar results were obtained when the investigators used SS-OCT to map regional choroidal thicknesses of the entire macula.^55^ The group found that SS-OCT significantly improved visualization of the posterior choroid and sclera, compared to conventional 840 nm OCT. To verify this finding, the same investigators quantitatively compared results obtained by SS-OCT and EDI-OCT in measuring retinal and choroidal thicknesses.^56^ Intersystem reproducibility was evaluated based on ICCs, which was approximately 0.661 for retinal thickness and 0.921 for choroidal thickness. The study also reported strong interexaminer reproducibility (ICC = 0.630 for retinal thickness, ICC = 0.912 for choroidal thickness) and strong intervisit reproducibility (ICC = 0.504 for retinal thickness, 0.893 for choroidal thickness) using SS-OCT. Applying these baseline results to the context of glaucoma, Usui et al^39^ measured choroidal thickness in highly myopic eyes with NTG. They determined that choroidal thickness in highly myopic NTG is considerably thinner than in myopic subjects without glaucoma, at the superior (p < 0.05), superotemporal (p < 0.01), temporal (p < 0.01) and inferotemporal (p < 0.05) regions around the optic disk and at the fovea (p < 0.001). Choroidal thinning may contribute to the disease process in glaucoma, by impeding choroid circulation and thus reducing perfusion of the LC.^57-62^ The thinning itself may result from mechanical stretching of the sclera, particularly in myopic eyes with already thin sclera, which may subsequently damage the LC and optic nerve.^57-62^ Therefore, accurate measurement of choroidal thickness, made possible through SS-OCT imaging, may provide useful information for evaluation of glaucoma.

Besides improving visualization of the choroid, SS-OCT promises to enable more accurate evaluation of the LC itself. We used the prototype SS-OCT system (Topcon Inc., Tokyo, Japan). This SS-OCT instrument has a scan speed of 100.000 Hz operated at the 1 μm wavelength region. It uses a wavelength-sweeping laser with a tuning range of approximately 100 nm as light source and has a center wavelength of 1050 nm, yielding approximately 8 μm axial resolution in tissue. These features allow deep penetration of posterior ocular structures. We used different scan protocols for evaluation of the lamina cribrosa. First, a 3D imaging data set was acquired with a 6 × 6 mm raster scan centered on the optic disk. The scans were composed of 256 B-scans each composed of 256 A-scans (total 65,536 axial scans/ volume) with an acquisition time of 1 second. Second, a 12-line radial scan, centered on the optic disk with a resolution of 1,024 and a scan count of 48 was acquired. All scan protocols used the choroidal reference position. The centering of scans was achieved by an internal-fixation and confirmed by a fundus camera integrated in the instrument.

**Fig. 1:**
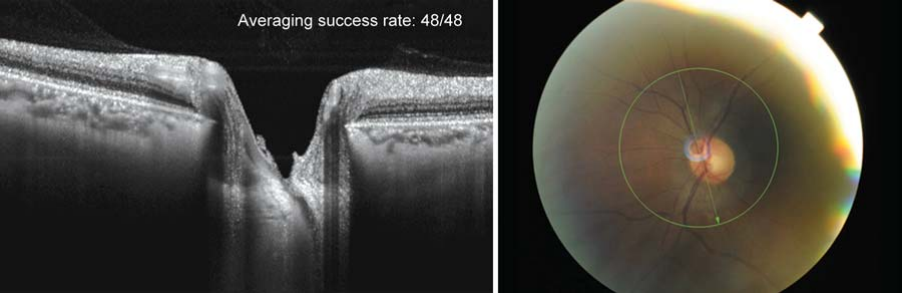
SS-OCT scan cuts through the main vascular trunk (fundus photo) with the central retinal vein visible as it cuts through the LC. Anterior and posterior border of the LC are visible throughout the entire optic canal opening including beneath the neuroretinal and scleral rim and indicated through hyporeflective openings with retinal nerve bundles passing through

**Fig. 2:**
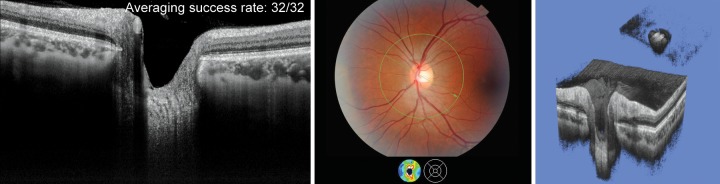
Fundus photo (center) and 2D radial scan of a healthy eye with excellent visualization of choroid (right) and anterior and posterior borders of the LC. 3D SS-OCT scan of the entire optic nerve head complex (right). The cropping function allows the user to slice the images and study morphological microstructures. A detailed dissection of the LC allows a detailed study of this structure (inset upper right)

**Fig. 3:**
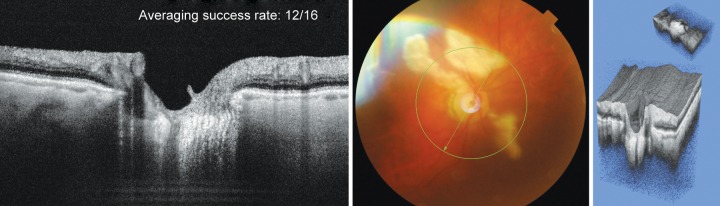
Example of 3D rendering of deep optic nerve structures in an eye with suspect glaucoma. The cropping function has eliminated all structures in order to obtain an isolated rendering of the LC (inset upper right)

**Fig. 4:**
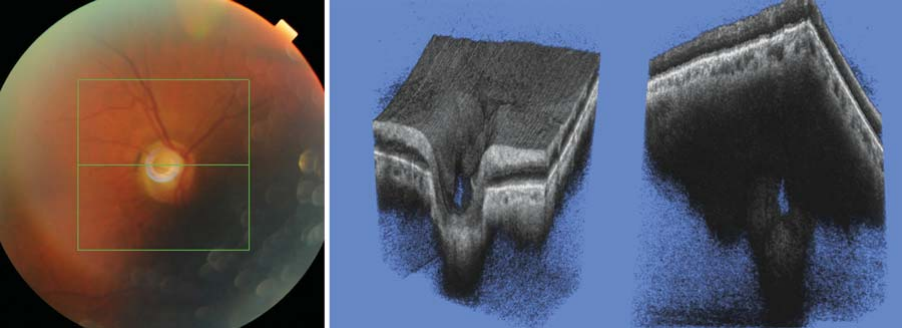
Glaucomatous eye. 3D SS-OCT show the presence of a large defect in the superotemporal quadrant of the optic nerve, which

**Fig. 5:**
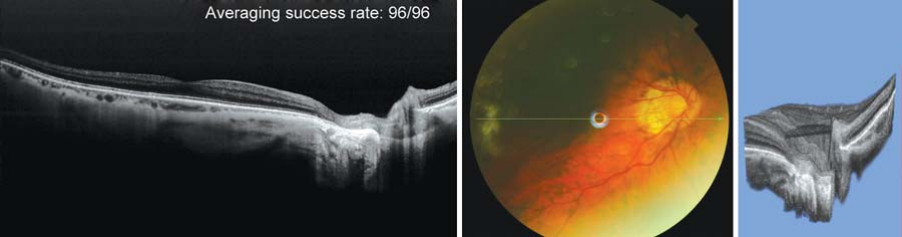
Highly myopic eye (—10.0 D) with established open-angle glaucoma and large temporal peripapillary atrophy. SS-OCT 12 mm line scan shows large reorganization of retinal anatomy in the PPA area including absence of retinal pigment epithelium (RPE) and Bruch’s membrane. In addition to the choroid, the sclera and emissarial vessels can be visualized in detail. 3D rendering was done at the level of the deepest fall of retinal nerve fibers through absent RPE and Bruch’s membrane support

Figures 1 to 5 provide examples of SS-OCT visualization of the LC and deep optic nerve head structures.

does not seem to be an artifact due to vessel shadowing

## CONCLUSION

SD/EDI-OCT and SS-OCT both enable visualization of the lamina cribrosa. SS-OCT may have some advantages for this use. With a more powerful imaging tool, the methodologies of previously described work can be re-applied and then allow for more accurate characterization of this important structure in the pathogenesis of glaucoma.
